# Eliminating Mother-to-Child Transmission of HIV by 2030: 5 Strategies to Ensure Continued Progress

**DOI:** 10.9745/GHSP-D-17-00097

**Published:** 2018-06-27

**Authors:** Alexandra C. Vrazo, David Sullivan, Benjamin Ryan Phelps

**Affiliations:** a Office of HIV/AIDS, United States Agency for International Development, Washington, DC, USA.

## Abstract

To keep up momentum in preventing mother-to-child transmission we propose: (1) advocating for greater political and financial commitment; (2) targeting high-risk populations such as adolescent girls and young women; (3) implementing novel service delivery models such as community treatment groups; (4) performing regular viral load monitoring during pregnancy and postpartum to ensure suppression before delivery and during breastfeeding; and (5) harnessing technology in monitoring and evaluation and HIV diagnostics.

## ENORMOUS PROGRESS IN PMTCT

Over the last 3 decades, prevention of mother-to-child transmission (PMTCT) of HIV programs have been at the forefront of HIV care and treatment innovation. The Global Plan Towards Eliminating New HIV Infections Among Children by 2015 and Keeping Their Mothers Alive, launched in 2011, set a series of ambitious targets for 2015 including the reduction of new HIV infections among children by 90% and AIDS-related maternal mortality by 50% from 2009 through 2015.^1^ The successful global movement to start all pregnant and breastfeeding women on antiretroviral therapy (ART) regardless of CD4 T-cell count or clinical staging (known as Option B+)^2^^,^^3^ set the stage for the World Health Organization's (WHO's) aptly named “Treat All” guidelines, which eliminated many long-standing barriers to HIV treatment.^4^ Meanwhile, data from several clinical trials—PROMISE, TEMPRANO, START, and HPTN 052—further demonstrated how outcomes improve with ART initiation at any CD4 T-cell count.^5^^–^^8^ These studies demonstrated lower morbidity and mortality, increased linkages to care, faster immune system reconstitution, and decreased HIV transmission in those starting ART sooner. The rapid scale up of Option B+ to more than 21 countries has demonstrated that programs designed to test and then quickly start treatment in all pregnant and breastfeeding women with HIV lead to increased enrollment, infections averted, and lives saved.^9^ The elimination of mother-to-child transmission of HIV in Armenia, Belarus, the Caribbean, Cuba, and Thailand is encouraging and historic. This multinational milestone provides a clear example of how a strategic approach can succeed when access to services is coupled with strong public health infrastructure, committed political leadership, coordinated engagement of multiple partners, sufficient funding, and a robust monitoring system.^10^^,^^11^

The increasing awareness of the ART regimens and program strategies that best prevent vertical transmission of HIV has led to a dramatic increase in access to ART among pregnant and breastfeeding women over the last decade. Global ART coverage is now estimated at 77% of pregnant women living with HIV, while ART coverage of all people living with HIV is lower, at approximately 53%. In 22 of the countries with the highest HIV burden—which together account for 90% of the global unmet PMTCT need—ART coverage has almost doubled for pregnant women, with 7 of these countries reaching 90% ART coverage rates among pregnant women living with HIV.^12^ Moreover, mother-to-infant transmission rates are now below 5% in several countries, including Ethiopia, South Africa, and Tanzania, moving toward the criteria for elimination of MTCT, defined in part by lowering new HIV cases to fewer than 50 per 100,000 live births.^9^

These advances in PMTCT may signal to some that the mother-to-child transmission battle is won, or soon will be. This is unfortunately not the case. An estimated 90% of the new annual incident infections in children under 15 are still due to mother-to-child transmission, likely due to undiagnosed incident infection during the pregnancy or breastfeeding period,^13^ and an estimated 2 million additional children under 15 will be living with HIV by 2020.^14^ To eliminate new HIV infections among children by 2030, we will need to build upon the successes and investments that have carried us this far. Below, we outline 5 actionable strategies that will consolidate the gains earned by PMTCT programs and help avoid reversals in progress already made.

## 5 STRATEGIES TO PROTECT AND BUILD ON PMTCT GAINS

### Strategy #1: Advocate for Intensified Political and Financial Commitment for PMTCT

The key take-home message from this article is this: Providers, policy makers, programmers, and communities must continue to advocate strongly for PMTCT, a platform that cost effectively saves 2 lives simultaneously (mother and infant) while preventing HIV and promoting family health. After the much-needed initial scale up of funding for global PMTCT programming and achievements in the earlier years of this decade, both funding and progress have slowed. The year 2015 saw the first global decrease in PMTCT funding since 2010, and further cuts are planned.^15^ As an example, PMTCT funding declined by 65% between 2013 and 2017 within the U.S. President's Emergency Plan for AIDS Relief (PEPFAR).^16^ While progress in antiretroviral (ARV) coverage for pregnant women showed rapid global scale up in the early part of the decade, increasing from the previous year by 17% in 2011 and by 13% in 2012, progress has slowed more recently, increasing by just 1.4% and 2.7% in 2016 and 2017, respectively.^9^

Providers, policy makers, programmers, and communities must continue to advocate strongly for PMTCT, a platform that saves the lives of both mother and infant while preventing HIV and promoting family health.

Advocates for sustaining PMTCT funding must frequently remind policy makers that PMTCT is cost-effective and protects not just 1 but 2 lives.^17^^–^^19^ Besides keeping mothers well, PMTCT protects HIV-exposed children from infection and decreases infant morbidity and mortality, thus reducing the need for infant ART and limiting HIV resistance.^20^^,^^21^ Leveraging the PMTCT platform to improve access to services to improve the health of mothers, infants, and children living with or at risk from HIV infection can help extend services efficiently in an era of increasingly limited resources. Family planning, nutrition, and maternal, neonatal, and child health services are often integrated, such as combining visits for immunizations with infant virological tests for HIV during the breastfeeding period.^22^^,^^23^ Other PMCT services can be integrated into immunization platforms, including rapid HIV testing of mothers who may have seroconverted during pregnancy, of those who did not come to antenatal care, or of those who did not receive an HIV test at antenatal care. Service integration is feasible across a variety of contexts, both in health facilities and in community settings, and can help protect recent hard-fought gains in child survival.^24^^,^^25^ With family planning, modeling exercises have shown that simple interventions such as the inclusion of contraceptives as part of the PMTCT package can have a greater impact on reducing the number of infants born with HIV infection than ARV drugs for PMTCT alone.^26^^,^^27^

Finally, PMTCT platforms can catalyze access to other HIV services, including HIV case finding for men and siblings through family-based models. Successful family-based interventions, such as “MTCT-Plus,” enrolled family members into care and treatment in countries including Côte d'Ivoire, Kenya, Malawi, and Thailand.^28^^–^^31^ MTCT-Plus is a comprehensive approach, linking PMTCT programs with HIV-specific primary and preventive care for the whole family.^32^ Conceptualized as a suite of services reaching entire families, the cost-effectiveness of PMTCT is compelling: Averting 90% of new pediatric infections alone would significantly reduce expenditures on pediatric care and treatment by country governments and donors. In addition to averting thousands of pediatric infections, a 90% decrease in PEPFAR's pediatric care and treatment costs could represent over $100 million in annual savings (based on PEPFAR planned expenditures in fiscal year 2017).

MTCT-Plus provides integrated services for the entire family, including clinical care and prevention, nutrition, family planning, counseling, and other supportive care, in both clinical and community settings.

### Strategy #2: Continue Focused Innovation to Target High-Risk Populations

PMTCT program innovation ushered in an era of “Treat All” and family-based care. Building on PMTCT programs that have redefined the HIV landscape, future innovations in PMTCT policy frameworks and program design must sensitively target populations that bear the highest burden of the epidemic, including adolescent girls and young women ages 10–24 and female sex workers.

**Adolescent girls and young women.** The “youth bulge” in Africa, where 61% of the population is under the age of 24, has made adolescent girls and young women a major at-risk population for HIV infection,^33^ where they now account for nearly 75% of new HIV infections in sub-Saharan Africa.^34^ Besides new incident infections, a substantial number of new HIV-positive diagnoses are made in perinatally infected adolescents.^35^^,^^36^ Together, these new and previously undiagnosed adolescent infections could represent a large contribution to new infant infections.

**Female sex workers.** HIV prevalence is an estimated 13.5 times higher among female sex workers than the general female population.^37^ The majority of female sex workers worldwide are mothers, and PMTCT platforms represent a unique opportunity to reach and retain these women and their infants in HIV care.^38^^–^^40^ However, due to criminalization, stigma, discrimination, and violence, access to respectful antenatal and postnatal care remains elusive for them, and poor HIV-related outcomes for mothers who identify as female sex workers and their infants are sadly all too common.^39^^,^^41^^–^^43^ Programmers can use innovative approaches such as differentiated service delivery models that simplify and adapt HIV services to better meet the needs of people while reducing burdens on the health system^44^ and stigma measurement tools for key populations^45^ to inform the design of respectful, sensitive, and stigma- and discrimination-free services to ensure that pregnant and breastfeeding female sex workers are not marginalized from PMTCT programs. Since a substantial proportion of incident HIV infections will occur in high-risk women who are pregnant and breastfeeding, to truly eliminate mother-to-child transmission and to achieve the 90-90-90 HIV treatment goals (by 2020, 90% of all people living with HIV will know their HIV status, 90% of all those diagnosed with HIV will receive ART, and 90% of those on ART will have viral suppression), it will be critical to incorporate periodic HIV retesting and PMTCT programming into both adolescent- and female sex worker-friendly sexual and reproductive health services.

### Strategy #3: Refine Novel Service Delivery Modalities for the PMTCT Context

Novel HIV service delivery approaches are crucial to reducing the burden on health facilities in the era of universal treatment, and programmers must advocate that pregnant and breastfeeding women living with HIV are included in these approaches. WHO recently outlined key considerations for promoting differentiated service delivery for pregnant and breastfeeding women living with HIV.^44^ Under a differentiated service delivery model, people living with HIV who are clinically stable on treatment would need to make less frequent clinic visits than people who are ill and need intensive follow-up. As delivery models evolve to incorporate these considerations specifically for pregnant and breastfeeding women living with HIV, the package of PMTCT services should be deliberately developed to maximize the advantages of these models. Numerous studies have reported on the barriers for pregnant and breastfeeding women—including travel time, cost, and disclosure—that impact both access to and retention in care.^46^^–^^51^ Many of these barriers are being addressed by bringing services closer to where women live. For example, in South Africa, almost 90,000 stable individuals belong to community ART groups implemented by Médecins Sans Frontières.^52^ These groups have promoted adherence among stable patients with enhanced viral load suppression and higher CD4 T-cell counts, and they have also improved linkages to other supportive services and retention in care.^53^^,^^54^ Appointment spacing and fast-track drug refills have also saved costs and increased retention by reducing clinical visits, the amount of time spent at the clinic, and staff workloads.^55^ Expanding the use of proven lay cadre platforms, such as mentor mothers and patient advocates, is another strategy to bring differentiated care models to pregnant and breastfeeding women.^25^^,^^56^^,^^57^

Recent WHO guidance for differentiated service delivery for pregnant or breastfeeding women highlights distinct considerations for clinically stable women who are accessing ART care at conception compared with women initiating ART during pregnancy or breastfeeding.

During development of differentiated models of ART delivery, it will be crucial for programmers to address the distinct needs of women in the pregnancy and breastfeeding periods, and to consider integration with maternal, neonatal, and child health services, including child health visits. To ensure increased uptake and scale up of the recent WHO considerations for differentiated service delivery in PMTCT programs, programmers should seek out opportunities to innovate and to publicize any resulting successes.

### Strategy #4: Recognize the Importance of Viral Load Monitoring During Pregnancy and Postpartum

Despite the promise held by new ARV formulations like dolutegravir for pregnant and breastfeeding women, current first-line ARV regimens have great potential to eliminate mother-to-child transmission as long as regular viral load monitoring is performed to identify challenges with ART adherence or clinical failure. The pregnancy and breastfeeding period represents a time-limited window in which reduced adherence and spikes in maternal viremia, even with ART usage, put the mother and infant at risk.^58^ However, there has been inadequate attention to viral load monitoring in pregnancy and the postpartum period. Recent considerations suggest that the frequency of viral load testing should be increased during pregnancy and breastfeeding, with the goal of achieving suppression before delivery and maintaining suppression during breastfeeding.^59^ Proposed interventions include viral load testing 4 weeks prior to delivery to set the stage for postpartum interventions in the event of high maternal viral load, including targeted birth testing and enhanced ARV birth prophylaxis.^59^^,^^60^

Much additional research remains on viral load monitoring in pregnancy and postpartum that cuts across clinical, programmatic, and behavioral areas. As a simple step, health cadres already in use in PMTCT programs, such as mentor mothers, can be employed to create demand for viral load monitoring; sensitize and inform mothers about the prolonged risk that incomplete adherence carries; and provide adherence support to mothers. For breastfeeding mothers, the clinical visits for the infant (including newborn visits, immunization visits, and well-child visits) can be leveraged as opportunities to provide access to maternal viral load testing throughout the period of infant exposure.

### Strategy #5: Harness Technology to Advance PMTCT's Impact

Technological advances in monitoring and evaluation and HIV diagnostics have the potential to further drive PMTCT program success. The Global Plan brought significant improvements to measuring PMTCT program outcomes, including low-tech longitudinal registers to track mother–infant pairs and an updated monitoring framework as countries moved to providing lifelong ART for women under Option B+.^61^^,^^62^ Despite these advances, ongoing core challenges to monitoring and evaluation include tracking women who move between general ART and PMTCT programs, monitoring maternal viral suppression, and assessing final infant HIV status. The individual medical record data needed to accurately determine these PMTCT outcomes are challenging to track in settings where health infrastructure is limited and electronic systems are not available.^63^ Several technologies have been used in PMTCT applications to complement paper-based systems to improve the collection and reporting of patient, facility, subnational, and national data to help programmers identify gaps in PMCT programming. Below are a few examples.

- **SMS-based reporting and messaging algorithms:** Short message service (SMS) technology has been used widely for mobile health (mHealth) initiatives. Many of these initiatives, including MomConnect in South Africa, have been used to provide bidirectional information to pregnant mothers and to health facility staff.^64^ Other SMS innovations in use for PMTCT include Uganda's nationwide weekly reporting system.^65^ Each registered PMTCT site reports weekly on 9 indicators by using mobile phones to text data, including on ARV and test kit stock-outs. In 2015, an average of 79% of the 1,687 registered PMTCT sites reported their data, which is received in a central database. This “small data” is analyzed and shared via an electronic dashboard to stakeholders including program managers, district health officials, and implementing partners to allow decisions on targeted technical assistance to sites. The online dashboard program is available publicly at http://dashboard.mets.or.ug, and a smartphone app is also available (B+Track). In Kenya, the HIV Infant Tracking System (HITSystem) uses algorithm-based computer and text messaging alerts. It was first demonstrated to improve the quality and efficiency of early infant diagnosis services in Kenyan hospitals and has been expanded to Malawi, Nigeria, and Tanzania.^66^ The tool links key stakeholders—clinicians, laboratory staff, and mothers—through web-based and SMS notifications with the goal of improving mother–infant retention in care and health outcomes. Lessons from SMS technologies have demonstrated that for successful use in PMTCT programs, early and sustained engagement with stakeholders, including mothers, health staff, and policy makers, is critical to effectively impact PMTCT outcomes.
- **Laboratory dashboards:** A key challenge in monitoring mother–infant health outcomes in PMTCT programs has been ineffectively used paper-based systems, which hamper the timely return of maternal viral load and infant virological test results, including early infant diagnosis. Lengthy turnaround times also contribute to delays in clinical management and represent a major challenge to managing viremia in mothers and infants living with HIV alike. Dashboards to support the review of national viral load and infant virological test program data have been created in Kenya (http://eid.nascop.org/) and Uganda (https://edash.cphluganda.org/) by integrating open-source laboratory information systems with cloud-based servers. These dashboards allow customizable data views for metrics at the national and subnational level, including viral load suppression rates for pregnant and breastfeeding mothers; infant positivity rate; infant ART initiation rate; early infant diagnosis and viral load turnaround times; and district- and facility-level data disaggregation. Providers and program managers can monitor progress in real time at the site and lab levels using these dashboards to complement patient-level data, and make adjustments to laboratory or clinical processes to ensure that women with high viral load and HIV-exposed infants receive care in a timely manner.
- **Novel point-of-care instruments:** One of the greatest challenges of the PMTCT cascade is the infant diagnostic test. In high-burden HIV settings, it can be very challenging to get samples from the patient to the laboratory and back, and then to communicate those results to the parent.^67^ Point-of-care (POC) instruments have the potential to be used at birth or later for infant diagnosis per recent WHO guidelines. Though POC instruments require unique maintenance, reagents, and provider training,^68^^,^^69^ field evaluations have shown that POC instruments are feasible for use in infant diagnosis,^70^^–^^73^ where they increase infant retention in care.^74^ When coupled with effective clinical and community linkage processes, POC infant testing promises to increase access to testing and improvements in retention among those diagnosed.^75^

Innovative approaches to reporting and visualizing program and laboratory data online have helped PMTCT programs track results in near real-time in Kenya, Malawi, Nigeria, Tanzania, and Uganda.

Integration of POC instruments into the conventional laboratory diagnostic network and coordination of procurements and trainings are critical considerations for planners to ensure POC technology adds value to PMTCT programs for infant diagnosis.

## CONCLUSION

PMTCT programs have made significant strides over the last 2 decades, but global funding for HIV is in decline while new pediatric infections persist. To continue to advance PMTCT programs, advocates, public health practitioners, and policy makers have promising options, including the ones outlined in this article. Strategies such as these will help ensure continued progress toward the elimination mother-to-child transmission of HIV, one of the key global health challenges of our time.

## Notes

**Peer Reviewed**

**Funding:** This publication was supported by the U.S. President's Emergency Plan for AIDS Relief through the United States Agency for International Development.

## References

[1] Joint United Nations Programme on HIV/AIDS (UNAIDS). On the Fast-Track to an AIDS-Free Generation. Geneva: UNAIDS; 2016. http://www.unaids.org/en/resources/documents/2016/GlobalPlan2016. Accessed May 8, 2019.

[2] Fasawe O Avila C Shaffer N. Cost-effectiveness analysis of Option B+ for HIV prevention and treatment of mothers and children in Malawi. PLoS One. 2013;8(3):e57778-e57778. 10.1371/journal.pone.0057778. 23554867 PMC3595266

[3] Granich RM Gilks CF Dye C De Cock KM Williams BG. Universal voluntary HIV testing with immediate antiretroviral therapy as a strategy for elimination of HIV transmission: a mathematical model. Lancet. 2009;373(9657):48–57. 10.1016/S0140-6736(08)61697-9. 19038438

[4] World Health Organization (WHO). Guideline on When to Start Antiretroviral Therapy and on Pre-exposure Prophylaxis for HIV. Geneva: WHO; 2015. http://www.who.int/hiv/pub/guidelines/earlyrelease-arv/en/. Accessed May 8, 2018. 26598776

[5] Danel C Moh R Gabillard D; TEMPRANO ANRS 12136 Study Group. A trial of early antiretrovirals and isoniazid preventive therapy in Africa. N Engl J Med. 2015;373(9):808–822. 10.1056/NEJMoa1507198. 26193126

[6] Lundgren JD Babiker AG Gordin F; INSIGHT START Study Group. Initiation of antiretroviral therapy in early asymptomatic HIV infection. N Engl J Med. 2015;373(9):795–807. 10.1056/NEJMoa1506816. 26192873 PMC4569751

[7] Cohen MS Chen YQ McCauley M Gamble T Hosseinipour M Kumarasamy N. Final results of the HPTN 052 randomized controlled trial: antiretroviral therapy prevents HIV transmission. J Int AIDS Soc. 2015;18(suppl 4):20479. 10.7448/IAS.18.5.20479 26205488 PMC4513179

[8] Fowler MG Qin M Fiscus SA. PROMISE: efficacy and safety of 2 strategies to prevent perinatal HIV transmission. Presented at: Conference on Retroviruses and Opportunistic Infections (CROI); February 23–26, 2015; Seattle, Washington. http://www.croiconference.org/sessions/promise-efficacy-and-safety-2-strategies-prevent-perinatal-hiv-transmission/. Accessed May 8, 2018.

[9] UNAIDS AIDSinfo. Geneva: Joint United Nations Programme on HIV/AIDS (UNAIDS). http://aidsinfo.unaids.org/. Accessed October 22, 2017.

[10] WHO validates countries' elimination of mother-to-child transmission of HIV and syphilis [statement]. Geneva: World Health Organization; June 8, 2016. http://www.who.int/en/news-room/detail/08-06-2016-who-validates-countries-elimination-of-mother-to-child-transmission-of-hiv-and-syphilis. Accessed May 14, 2018.

[11] Six Caribbean territories and states eliminate mother-to-child transmission of HIV and syphilis [news release]. Washington, DC: Pan American Health Organization; last updated December 6, 2017. https://www.paho.org/hq/index.php?option=com_content&view=article&id=13961&;Itemid=1926&lang=en. Accessed May 14, 2018.

[12] Prevention of mother-to-child transmission (PMTCT): situation and trends. Global Health Observatory (GHO) data. World Health Organization website. http://www.who.int/gho/hiv/epidemic_response/PMTCT_text/en/. Accessed May 14 2018.

[13] Ciaranello AL Perez F Keatinge J. What will it take to eliminate pediatric HIV? Reaching WHO target rates of mother-to-child HIV transmission in Zimbabwe: a model-based analysis. PLoS Med. 2012;9(1):e1001156-e1001156. 10.1371/journal.pmed.1001156. 22253579 PMC3254654

[14] Penazzato M Bendaud V Nelson L Stover J Mahy M. Estimating future trends in paediatric HIV. AIDS. 2014;28(suppl 4):S445–S451. 10.1097/QAD.0000000000000481. 25409099 PMC4247271

[15] Kates J Wexler A Lief E. Financing the Response to HIV in Low- and Middle-Income Countries: International Assistance from Donor Governments in 2015. Geneva: UNAIDS and the Henry J. Kaiser Family Foundation; 2015. http://www.unaids.org/sites/default/files/media_asset/financing-the-response-to-HIV-in-low-and-middle-income-countries_en.pdf. Accessed May 8, 2018.

[16] PEPFAR Dashboards: Planned Funding, 2016. Washington, DC: U.S. President's Emergency AIDS Relief (PEPFAR). https://data.pepfar.net/country/funding. Accessed July 7, 2017.

[17] Johri M Ako-Arrey D. The cost-effectiveness of preventing mother-to-child transmission of HIV in low- and middle-income countries: systematic review. Cost Eff Resour Alloc. 2011;9(1):3-3. 10.1186/1478-7547-9-3. 21306625 PMC3045936

[18] Kuznik A Lamorde M Hermans S. Evaluating the cost-effectiveness of combination antiretroviral therapy for the prevention of mother-to-child transmission of HIV in Uganda. Bull World Health Organ. 2012;90(8):595–603. 10.2471/BLT.11.095430. 22893743 PMC3417786

[19] Bollinger L Adebiyi A. Cost-Effectiveness of Integrating PMTCT and MNCH Services: An Application of the LiST Model for Malawi, Mozambique, and Uganda. DHS Occasional Paper No. 7. Calverton, MD: ICF International; 2013. https://dhsprogram.com/publications/publication-op7-occasional-papers.cfm. Accessed May 8, 2018.

[20] Luzuriaga K. Early combination antiretroviral therapy limits HIV-1 persistence in children. Annu Rev Med. 2016;67(1):201–213. 10.1146/annurev-med-091114-111159. 26768239

[21] Becquet R Marston M Dabis F; UNAIDS Child Survival Group. Children who acquire HIV infection perinatally are at higher risk of early death than those acquiring infection through breastmilk: a meta-analysis. PLoS One. 2012;7(2):e28510-e28510. 10.1371/journal.pone.0028510. 22383946 PMC3285615

[22] Chamla DD Essajee S Young M. Integration of HIV in child survival platforms: a novel programmatic pathway towards the 909090 targets. J Int AIDS Soc. 2015;18(suppl 6):20250. 10.7448/IAS.18.7.20250. 26639111 PMC4670840

[23] United Nations Children's Fund (UNICEF). The Double Dividend: Action to Improve Survival of HIV Exposed Children in the Era of eMTCT and Renewed Child Survival Campaigns. New York: UNICEF; 2013. https://www.unicef.org/aids/files/Action_Framework_Final(1).pdf. Accessed May 8, 2018.

[24] Lindegren ML Kennedy CE Bain-Brickley D. Integration of HIV/AIDS services with maternal, neonatal and child health, nutrition, and family planning services. Cochrane Database Syst Rev. 2012;(9):CD010119. 10.1002/14651858.CD010119. 22972150 PMC12551653

[25] Rotheram-Borus MJ Tomlinson M le Roux IM. A cluster randomised controlled effectiveness trial evaluating perinatal home visiting among South African mothers/infants. PLoS One. 2014;9(10):e105934-e105934. 10.1371/journal.pone.0105934. 25340337 PMC4207699

[26] Reynolds HW Janowitz B Homan R Johnson L. The value of contraception to prevent perinatal HIV transmission. Sex Transm Dis. 2006;33(6):350–356. 10.1097/01.olq.0000194602.01058.e1. 16505747

[27] Halperin DT Stover J Reynolds HW. Benefits and costs of expanding access to family planning programs to women living with HIV. AIDS. 2009;23(suppl 1):S123–S130. 10.1097/01.aids.0000363785.73450.5a. 20081384

[28] Betancourt TS Abrams EJ McBain R Fawzi M. Family-centred approaches to the prevention of mother to child transmission of HIV. J Int AIDS Soc. 2010;13(suppl 2):S2. 10.1186/1758-2652-13-S2-S2. 20573284 PMC2890971

[29] Tonwe-Gold B Ekouevi DK Bosse CA. Implementing family-focused HIV care and treatment: the first 2 years' experience of the mother-to-child transmission-plus program in Abidjan, Côte dIvoire. Trop Med Int Health. 2009;14(2):204–212. 10.1111/j.1365-3156.2008.02182.x. 19236666 PMC2793410

[30] Toro PL Katyal M Carter RJ; MTCT-Plus Initiative. Initiation of antiretroviral therapy among pregnant women in resource-limited countries: CD4+ cell count response and program retention. AIDS. 2010;24(4):515–524. 10.1097/QAD.0b013e3283350ecd. 19996939

[31] Myer L Abrams EJ Zhang Y Duong J El-Sadr WM Carter RJ. Family matters: co-enrollment of family members into care is associated with improved outcomes for HIV-infected women initiating antiretroviral therapy. J Acquir Immune Defic Syndr. 2014;67(suppl 4):S243–S249. 10.1097/QAI.0000000000000379. 25436824 PMC4252141

[32] Rabkin M El Sadr WM. Saving Mothers, Saving Families: The MTCT-Plus Initiative: Case Study. Geneva: World Health Organization; 2003. http://www.who.int/hiv/pub/prev_care/en/Saving_Mothers_E.pdf. Accessed May 8, 2018.

[33] United Nations (UN), Department of Economics and Social Affairs, Population Division. World Population Prospects–2017 Revision. New York: UN; 2017. https://esa.un.org/unpd/wpp/. Accessed May 8, 2018.

[34] The Global Fund. Adolescent Girls and Young Women in High-HIV Burden Settings: Technical Brief. Geneva: The Global Fund; 2017. https://www.theglobalfund.org/media/4576/core_adolescentgirlsandyoungwomen_technicalbrief_en.pdf. Accessed May 8, 2018.

[35] Ferrand RA Munaiwa L Matsekete J. Undiagnosed HIV infection among adolescents seeking primary health care in Zimbabwe. Clin Infect Dis. 2010;51(7):844–851. 10.1086/656361. 20804412 PMC4578231

[36] Simms V Dauya E Dakshina S. Community burden of undiagnosed HIV infection among adolescents in Zimbabwe following primary healthcare-based provider-initiated HIV testing and counselling: a cross-sectional survey. PLoS Med. 2017;14(7):e1002360. 10.1371/journal.pmed.1002360. 28742829 PMC5526522

[37] Kerrigan D Wirtz A Baral S. The Global HIV Epidemics Among Sex Workers. Washington, DC: World Bank; 2013. https://openknowledge.worldbank.org/handle/10986/12217. Accessed May 8, 2018.

[38] Maheu-Giroux M Vesga JF Diabaté S. Population-level impact of an accelerated HIV response plan to reach the UNAIDS 90-90090 target in Côte d'Ivoire: insights from mathematical modeling. PLoS Med. 2017;14(6):e1002321. 10.1371/journal.pmed.1002321. 28617810 PMC5472267

[39] Papworth E Schwartz S Ky-Zerbo O. Mothers who sell sex: a potential paradigm for integrated HIV, sexual, and reproductive health interventions among women at high risk of HIV in Burkina Faso. J Acquir Immune Defic Syndr. 2015;68(suppl 2):S154–S161. 10.1097/QAI.0000000000000454. 25723980

[40] Schwartz S Papworth E Thiam-Niangoin M. An urgent need for integration of family planning services into HIV care: the high burden of unplanned pregnancy, termination of pregnancy, and limited contraception use among female sex workers in Côte d'Ivoire. J Acquir Immune Defic Syndr. 2015;68(suppl 2):S91–S98. 10.1097/QAI.0000000000000448. 25723996

[41] Lazarus L Deering KN Nabess R Gibson K Tyndall MW Shannon K. Occupational stigma as a primary barrier to health care for street-based sex workers in Canada. Cult Health Sex. 2012;14(2):139–150. 10.1080/13691058.2011.628411. 22084992 PMC3359131

[42] Willis B Welch K Onda S. Health of female sex workers and their children: a call for action. Lancet Glob Health. 2016;4(7):e438–e439. 10.1016/S2214-109X(16)30071-7. 27185469

[43] Miller J. From Shadows to Light: Advocacy for Children of HIV-Affected Key Populations. The Coalition for Children Affected by AIDS; 2016. https://aidsdatahub.org/shadows-light-advocacy-children-hiv-affected-key-populations-miller-j-2016. Accessed May 8, 2018.

[44] World Health Organization (WHO); U.S. Centers for Disease Control and Prevention (CDC); U.S. President's Emergency Plan for AIDS Relief (PEPFAR); U.S. Agency for International Development (USAID); International AIDS Society (IAS). Key Considerations for Differentiated Antiretroviral Therapy Delivery for Specific Populations: Children, Adolescents, Pregnant and Breastfeeding Women and Key Populations. Geneva: WHO; 2017. http://origin.who.int/hiv/pub/arv/hiv-differentiated-care-models-key-populations/en/. Accessed May 9, 2018.

[45] Stahlman S Hargreaves JR Sprague L Stangl AL Baral SD. Measuring sexual behavior stigma to inform effective HIV prevention and treatment programs for key populations. JMIR Public Health Surveil. 2017;3(2):e23. 28446420 10.2196/publichealth.7334 PMC5425775

[46] Gourlay A Birdthistle I Mburu G Iorpenda K Wringe A. Barriers and facilitating factors to the uptake of antiretroviral drugs for prevention of mother-to-child transmission of HIV in sub-Saharan Africa: a systematic review. J Int AIDS Soc. 2013;16(1):18588-18588. 10.7448/IAS.16.1.18588. 23870277 PMC3717402

[47] Panditrao M Darak S Jori V Kulkarni S Kulkarni V. Barriers associated with the utilization of continued care among HIV-infected women who had previously enrolled in a private sector PMTCT program in Maharashtra, India. AIDS Care. 2015;27(5):642–648. 10.1080/09540121.2014.990868. 25559362

[48] Duff P Kipp W Wild TC Rubaale T Okech-Ojony J. Barriers to accessing highly active antiretroviral therapy by HIV-positive women attending an antenatal clinic in a regional hospital in western Uganda. J Int AIDS Soc. 2010;13(1):37-37. 10.1186/1758-2652-13-37. 20863399 PMC2954932

[49] Matheson R Moses-Burton S Hsieh AC. Fundamental concerns of women living with HIV around the implementation of Option B+. J Int AIDS Soc. 2015;18(suppl 5):20286. 10.7448/IAS.18.6.20286. 26643459 PMC4672458

[50] Colvin CJ Konopka S Chalker JC. A systematic review of health system barriers and enablers for antiretroviral therapy (ART) for HIV-infected pregnant and postpartum women. PLoS One. 2014;9(10):e108150-e108150. 10.1371/journal.pone.0108150. 25303241 PMC4193745

[51] Kim MH Zhou A Mazenga A. Why did I stop? Barriers and facilitators to uptake and adherence to ART in Option B+ HIV care in Lilongwe, Malawi. PLoS One. 2016;11(2):e0149527. 10.1371/journal.pone.0149527. 26901563 PMC4762691

[52] Grimsrud A. Innovations in antiretroviral therapy delivery. Presented at: Conference on Retroviruses and Opportunistic Infections (CROI); February 22–26, 2016; Boston, MA. http://www.croiconference.org/sessions/innovations-antiretroviral-therapy-delivery. Accessed May 8, 2018.

[53] Wilkinson J Sharp L Cox V Cragg C Van Cutsem G Grimsrud A. Outcomes of patients enrolled in ART adherence clubs after viral resuppression. Presented at: Conference on Retroviruses and Opportunistic Infections (CROI); February 22–26, 2016; Boston, MA. http://www.croiconference.org/sessions/outcomes-patients-enrolled-art-adherence-clubs-after-viral-resuppression-0. Accessed May 8, 2018.

[54] Rasschaert F Decroo T Remartinez D. Adapting a community-based ART delivery model to the patient's needs: a mixed methods research in Tete, Mozambique. BMC Public Health. 2014;14:364–364. 10.1186/1471-2458-14-364. 24735550 PMC3990026

[55] Bemelmans M Baert S Goemaere E. Community-supported models of care for people on HIV treatment in sub-Saharan Africa. Trop Med Int Health. 2014;19(8):968–977. 10.1111/tmi.12332. 24889337

[56] Grimwood A Fatti G Mothibi E Malahlela M Shea J Eley B. Community adherence support improves programme retention in children on antiretroviral treatment: a multicentre cohort study in South Africa. J Int AIDS Soc. 2012;15(2):17381–17381. 10.7448/IAS.15.2.17381. 22713255 PMC3499784

[57] Shroufi A Mafara E Saint-Sauveur JF Taziwa F Viñoles MC. Mother to Mother (M2M) peer support for women in prevention of mother to child transmission (PMTCT) programmes: a qualitative study. PLoS One. 2013;8(6):e64717. 10.1371/journal.pone.0064717. 23755137 PMC3673995

[58] Tubiana R Le Chenadec J Rouzioux C. Factors associated with mother-to-child transmission of HIV-1 despite a maternal viral load <500 copies/ml at delivery: a case-control study nested in the French perinatal cohort (EPF-ANRS CO1). Clin Infect Dis. 2010;50(4):585–596. 10.1086/650005. 20070234

[59] Myer L Essajee S Broyles LN. Pregnant and breastfeeding women: a priority population for HIV viral load monitoring. PLoS Med. 2017;14(8):e1002375. 10.1371/journal.pmed.1002375. 28809929 PMC5557351

[60] World Health Organization (WHO). Consolidated Guidelines on the Use of Antiretroviral Drugs for Treating and Preventing HIV Infection: Recommendations for a Public Health Approach. 2nd ed. Geneva; WHO; 2016. http://www.who.int/hiv/pub/arv/arv-2016/en/. Accessed May 8, 2018. 27466667

[61] Radin AK Abutu AA Okwero MA. Confronting challenges in monitoring and evaluation: innovation in the context of the Global Plan Towards the Elimination of New HIV Infections Among Children by 2015 and Keeping Their Mothers Alive. J Acquir Immune Defic Syndr. 2017;75(suppl 1):S66–S75. 10.1097/QAI.0000000000001313. 28398999 PMC5407381

[62] Inter-agency Task Team on the Prevention and Treatment of HIV Infection in Pregnant Women, Mothers and Children (IATT). Monitoring & Evaluation Framework for Antiretroviral Treatment for Pregnant and Breastfeeding Women Living With HIV and Their Infants (IATT M&E Option B+ Framework). New York: U.S. Centers for Disease Control and Prevention, World Health Organization, and United Nations Children's Fund; 2015. https://www.childrenandaids.org/sites/default/files/2017-05/IATT-Framework-Monitoring-Evaluation-Framework-for-ART-Treat.pdf. Accessed May 9, 2018.

[63] Maru D Upreti S. To survive, health care needs small data to become adaptive. The Lancet Global Health Blog. May 12, 2017. http://globalhealth.thelancet.com/2017/05/12/survive-health-care-needs-small-data-become-adaptive. Accessed May 9, 2018.

[64] Barron P Pillay Y Fernandes A Sebidi J Allen R. The MomConnect mHealth initiative in South Africa: early impact on the supply side of MCH services. J Public Health Policy. 2016;37(suppl 2):201–212. 10.1057/s41271-016-0015-2. 27899795

[65] Porter LE Bouey PD Curtis S. Beyond indicators: advances in global HIV monitoring and evaluation during the PEPFAR era. J Acquir Immune Defic Syndr. 2012;60(suppl 3):S120–S126. 10.1097/QAI.0b013e31825cf345. 22797733

[66] Finocchario-Kessler S Gautney BJ Khamadi S; HITSystem Team. If you text them, they will come: using the HIV infant tracking system to improve early infant diagnosis quality and retention in Kenya. AIDS. 2014;28(suppl 3):S313–S321. 10.1097/QAD.0000000000000332. 24991904 PMC4226133

[67] Celletti F Cohn J. Using innovative technology to optimise early testing, care, and treatment for HIV-exposed infants. The Lancet Global Health Blog. February 5, 2016. http://globalhealth.thelancet.com/2016/02/05/using-innovative-technology-optimise-early-testing-care-and-treatment-hiv-exposed-infants. Accessed May 9, 2018.

[68] Habiyambere V Ford N Low-Beer D. Availability and use of HIV monitoring and early infant diagnosis technologies in WHO member states in 2011–2013: analysis of annual surveys at the facility level. PLoS Med. 2016;13(8):e1002088. 10.1371/journal.pmed.1002088. 27551917 PMC4995037

[69] Williams J Umaru F Edgil D Kuritsky J. Progress in harmonizing tiered HIV laboratory systems: challenges and opportunities in 8 African countries. Glob Health Sci Pract. 2016;4(3):467–480. 10.9745/GHSP-D-16-00004. 27688718 PMC5042701

[70] Jani IV Meggi B Mabunda N. Accurate early infant HIV diagnosis in primary health clinics using a point-of-care nucleic acid test. J Acquir Immune Defic Syndr. 2014;67(1):e1–e4. 10.1097/QAI.0000000000000250. 24933096

[71] Meggi B Bollinger T Mabunda N. Point-of-care p24 infant testing for HIV may increase patient identification despite low sensitivity. PLoS One. 2017;12(1):e0169497. 10.1371/journal.pone.0169497. 28060886 PMC5218410

[72] Ibrahim M Moyo S Mohammed T. Evaluation of the Cepheid HIV-1 Qual point-of-care test for HIV diagnosis at birth. Presented at: Conference on Retroviruses and Opportunistic Infections (CROI); February 13–16, 2017; Seattle, WA. http://www.croiconference.org/sessions/evaluation-cepheid-hiv-1-qual-point-care-test-hiv-diagnosis-birth. Accessed May 9, 2018.

[73] Sabi I Mahiga H Mgaya J. Evaluation of maternal and infant HIV point-of-care diagnostics at birth in Tanzania. Presented at: Conference on Retroviruses and Opportunistic Infections (CROI); February 13-16, 2017; Seattle, WA. http://www.croiconference.org/sessions/evaluation-maternal-and-infant-hiv-point-care-diagnostics-birth-tanzania. Accessed May 9, 2018.

[74] Jani IV Meggi B Loquiha O. Effect of point-of-care testing on antiretroviral-therapy initiation rates in infants. Presented at: Conference on Retroviruses and Opportunistic Infections (CROI); February 13–16, 2017; Seattle, WA. http://www.croiconference.org/sessions/effect-point-care-testing-antiretroviral-therapy-initiation-rates-infants. Accessed May 9, 2018.

[75] Dunning L Hsiao N Myer L. Point-of-care HIV early infant diagnosis: is test sensitivity everything? J Int AIDS Soc. 2015;18(1):20235. 10.7448/IAS.18.1.20235. 26344017 PMC4561293

